# Experimental Study on Residual Bending Strength of Corroded Reinforced Concrete Beam Based on Micromagnetic Sensor

**DOI:** 10.3390/s18082635

**Published:** 2018-08-11

**Authors:** Jianting Zhou, Junli Qiu, Yingxin Zhou, Yi Zhou, Runchuan Xia

**Affiliations:** 1 College of Civil Engineering, Chongqing Jiaotong University, Chongqing 400074, China; 622170086071@mails.cqjtu.edu.cn (J.Q.); rcxia@mails.cqjtu.edu.cn (R.X.); 2 Yunnan Wuyi Expressway Construction Command, Kunming 650000, China; zyx668@126.com; 3 Chongqing Yapai Bridge Engineering Quality Inspection Co., Ltd., Chongqing 401120, China; boatzy@163.com

**Keywords:** corroded reinforced concrete beam, bending strength, micromagnetic sensor, self-magnetic flux leakage, magnetic dipole model

## Abstract

This paper presents a nondestructive test method to evaluate the residual bending strength of corroded reinforced concrete beam by analyzing the self-magnetic flux leakage (SMFL) signals. The automatic scanning device was equipped with a micromagnetic sensor and sensor-based experimental details were introduced. Next, the theoretical formula of the normal component *H_S_*(*z*) of the SMFL signal that originated from the corroded region was derived based on the magnetic dipole model and the experimental results were discussed. The results indicate that the experimental data of *H_S_*(*z*) are consistent with the theoretical calculations, both location and extent of the steel bars corrosion can be qualitatively determined by using *H_S_*(*z*). The gradient *K* of *H_S_*(*z*) is approximately linearly related to the loss rate, *S*, of the bending strength, which can be used to evaluate the residual bending strength of the corroded reinforced concrete beam. This work lays the foundation for evaluating the residual bending strength of corroded reinforced concrete beams using the SMFL signal; the micromagnetic sensor is further applied to the civil engineering.

## 1. Introduction

Reinforced concrete structures are widely used in civil engineering due to their high bearing capacity, low cost, and easy construction. However, long-term exposure to the external aggressive environment, means that the reinforcement steel bars of the reinforced concrete structure will be corroded inevitably. The steel corrosion will lead to reduction of the steel bar cross-section, failure of the bond between the steel bar and the concrete, and deterioration of the concrete, which will ultimately weaken the bearing capacity and durability of the reinforced concrete structure [1,2,3]. Therefore, the measurement of corroded steel bar is crucial to determine the bearing capacity and durability of the reinforced concrete structures.

In the past, traditional electrochemical methods were used to measure corrosion of steel bars, but there are many limitations with these methods. In recent years, smart sensor-based methods that are useful to measure different defects (corrosion, cracks, etc.) of ferromagnetic materials (steel bars, etc.) and other materials have become increasingly popular, such as fiber optic coil winding [4], fiber Bragg grating [5,6], laser scanning technology [7], line scanning thermography (LST) [8,9], inductive thermography [10,11], optical excitation thermography [12], and other thermographic methods [13] such as acoustic emission and ultrasound testing using traditional and novel transducers [14,15]. Especially, the electromagnetic nondestructive test (NDT) including magnetic flux leakage (MFL) [16], metal magnetic memory (MMM) [17], magnetic acoustic emission (MAE) [18], magnetic Barkhausen noise (MBN) [19], and self-magnetic flux leakage (SMFL) [20] are widely used to measure corrosion and defects of ferromagnetic materials.

Based on the magnetic mechanical effect in the weak magnetic field, factors such as stress and defects within the ferromagnetic material will cause SMFL on its surface by orienting the magnetic domains, dislocating the lattices, and distorting the magnetic field lines [20,21]. Steel bars are a typical kind of ferromagnetic material; the corrosion of steel bars will reduce the bearing capacity and change the SMFL signals of the reinforced concrete structure at the same time. Based on this unified change, the relationship between the bearing capacity and the SMFL signals of corroded reinforced concrete structure can be established. Compared with MFL, SMFL has the advantages of rapid, effective, and simpler operation without any external excitation magnetic field required [22]. Liu [23,24,25] established a full electronic potential magneto-mechanical model of the ferromagnetic material and explored the relationship between magnetic memory signal and the stress concentration defects. Zhang [26,27] studied the relationship between the SMFL signals and the corrosion extent of corroded reinforced concrete. Zhang [28] investigated the fundamental relationship between corrosion rate and magnetic induction surrounding steel reinforcement. N. Polydorides [29] realized the magnetic induction tomography scanning of corroded reinforced concrete columns. Sun [30] had quantitatively studied the magnetic anomalies of reinforcement rods in bored in situ concrete piles for the first time and summarized their magnetic anomaly character. A. Orbe [31] proposed a magnetic scanning methodology to infer, nondestructively, the spatial dispersion of mechanical properties throughout the steel fiber reinforced concrete (SFRC) structure. H.-J Krause [32] developed a four-channel SQUID system based on magnetometers for detection of tendon ruptures in prestressed members of bridges. B.T. Fernandes [33] described a method of extracting positional information from images of steel bars embedded in concrete using a set of image preprocessing algorithms combined with a modified Hough transform. Chen [34] studied the corrosion of steel bars in reinforced concrete columns based on MFL. However, experimental study of the relationship between the bending strength and SMFL signals of corroded reinforced concrete beam is rarely reported.

In this paper, by detecting and analyzing the bending strength and SMFL signals of the corroded reinforced concrete beam, not only the location and extent of corrosion can be determined, but also the relationship between the bending strength and the SMFL signals. It will provide a rapid, effective, and simpler operation NDT method for the bending strength of corroded reinforced concrete beams.

## 2. Experimental Details and Theoretical Background

### 2.1. Experimental Details Based on Micromagnetic Sensor

**Figure 1:**
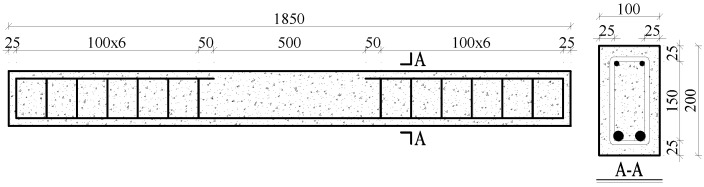
Dimensions of the tested beam (unit: mm).

**Figure 2:**
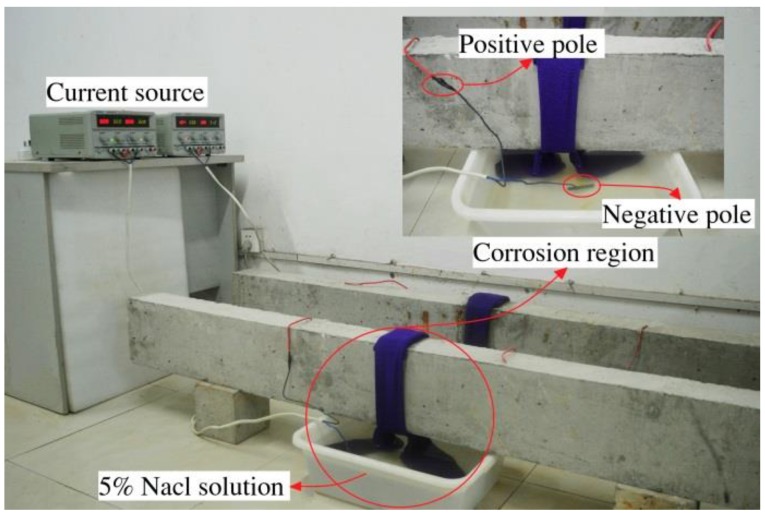
Layout of the corrosion device.

To carry out the experiment, 10 identical reinforced concrete tested beams, numbered 1–10, were prepared. In order to explore the relationship between the bending strength and the SMFL signals of the corroded reinforced concrete tested beams more intuitively, central sections (corrosion region) of the tested beams were only reinforced with two tensile steel bars. Ordinary Portland cement, coarse aggregate (stone), with a maximum size of 22 mm, water, and sand were used to mix concrete, the detailed material parameters and dimensional drawings of the tested beam are shown in Table 1 and Figure 1. An electrochemical method [35] was used to corrode steel bars of the tested beams, and the amount of corroded iron could be calculated according to the Faraday’s 1st Law, as expressed in Equation (1):

(1) $$\Delta m=\frac{M}{nF}\Delta Q=\frac{M}{nF}I\Delta t$$

where *Δm* is the amount of corroded iron, *M* is the molar mass of iron (Fe), *n* is the valence state of Fe^2+^, *F* is the Faraday constant, Δ*Q* is the quantity of electric charge, *I* is the direct current flow through steel bars, and Δ*t* is the corrosion time. The corrosion region is located at the midpoint of the tested beam with a width of approximately 15 cm, wrapped with a towel impregnated with a 5% sodium chloride solution and kept moist by the capillary action. The carbon rod placed in the solution is connected to the negative pole of the current source, and the steel bars are connected to the positive pole, forming a closed circuit for corrosion. Corrosion parameters of all tested beams are presented in Table 2, the layout of the corroding device is shown in Figure 2.

**Figure 3:**
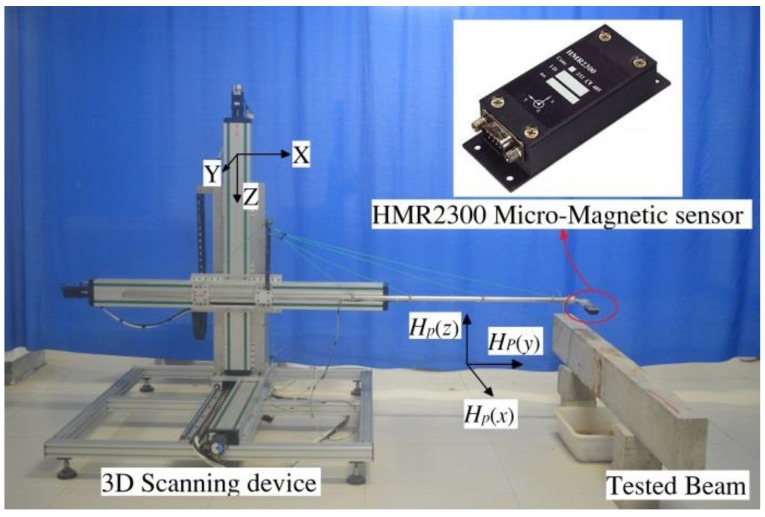
The 3D scanning device for magnetic field measurement.

**Figure 4:**
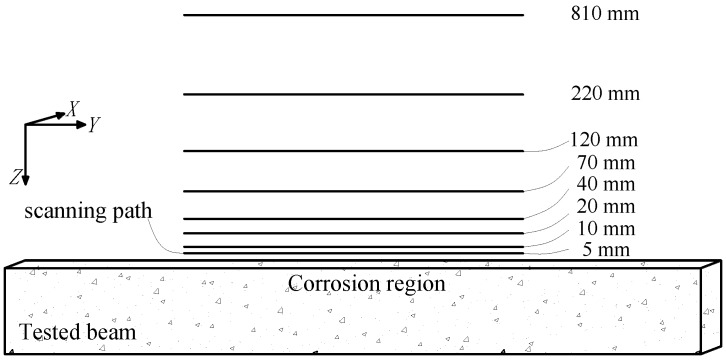
The scanning path of the tested beams to acquire self-magnetic flux leakage (SMFL) signals.

Each tested beam was corroded in a periodic manner until the corrosion was completed. After each period of corrosion was completed, the SMFL signals of the tested beam were measured and collected. The self-designed 3-dimensional (3D) device for SMFL signals acquisition based on the 3D mechanical displacement system and the high-precision micromagnetic sensor is shown in Figure 3. HMR2300 magnetometer (Honeywell International, Morristown, NJ, USA) was used as the micromagnetic sensor with a resolution to less than 70 μGs. The device was connected to a computer and can measure the 3D spatial magnetic signal with a controllable scanning speed and path, and then output a data file that contains *X*, *Y*, and *Z* coordinates and its corresponding magnetic signals components *H_P_*(*y*), *H_P_*(*x*), *H_P_*(*z*). The top surface midline of the tested beam is defined as the scanning path for SMFL signals measurement using the 3D scanning device, which is shown in Figure 4. In addition, Figure 4 also shows the different scanning lift-off heights (LFH) from 5 mm to 810 mm.

**Figure 5:**
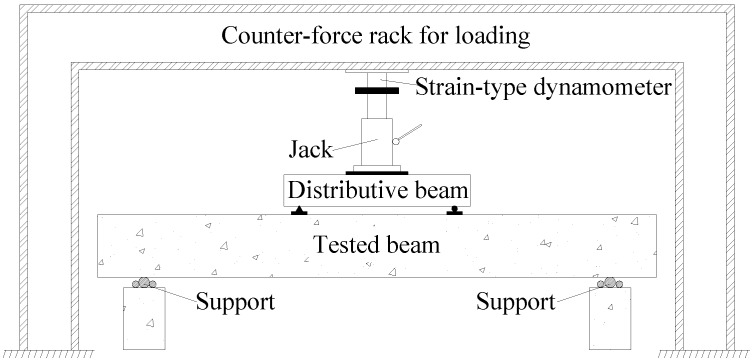
Layout of the “4-point” bending test.

All the tested beams were subjected to the “4-point” bending test after the corrosion and SMFL signals acquisition were all completed. The layout of the “4-point” bending test is shown in Figure 5.

### 2.2. Theoretical Background Based on Magnetic Dipole Model

**Figure 6:**
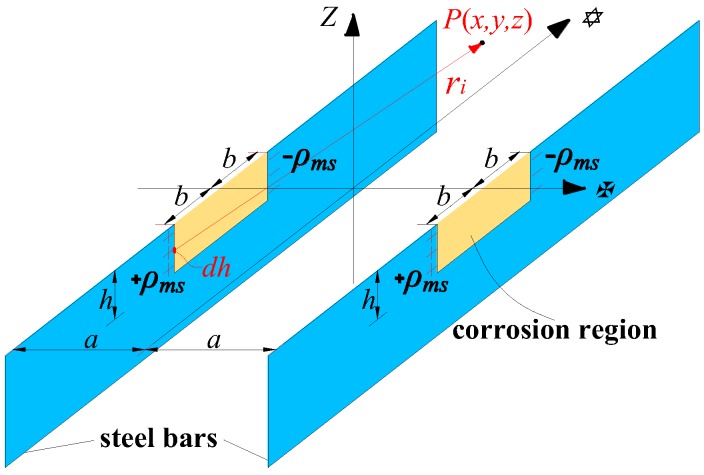
Calculated diagram based on the magnetic dipole model.

The equivalent magnetic dipole is a physical model commonly used in the theoretical study of the mechanism of SMFL signal of the ferromagnet [22,27,36]. According to the equivalent magnetic charge theory, the exterior magnetic field including SMFL would be considered to originated from the magnetic charge: *ρ* = −∇*M*. *M* is the magnetization satisfying *M* = (*μ*_r_ − 1) *H*_mL_, where *μ*_r_ is the relative magnetic permeability and the Weiss field *H*_mL_ is the effective field producing self-magnetization in the ferromgnet. Figure 6 shows the calculated diagram of the corroded steel bars of the tested beam based on the magnetic dipole model. The corrosion notch of the steel bar is assumed to a rectangular pit with dimensions of 2*b* × *h* and the distribution of charge density ±*ρ*_ms_ at the edges of the corroded region is considered as uniform for simplicity.

**Figure 7:**
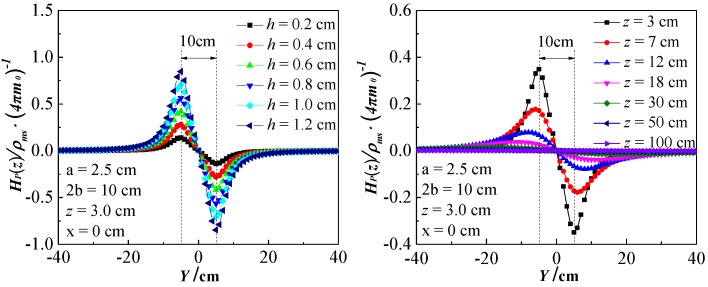
The theoretical calculation results of Equation (4).

Based on the calculated diagram shown in Figure 6, the surface SMFL signal at point *P*(*x*, *y*, *z*) due to these concentrated magnetic charges can be expressed as in Equation (2), and its normal component *dH_p_*(*z*) can be expressed as in Equation (3):

(2) $$\vec{dH_{P}}=\sum_{i=1}^{4}\vec{dH_{Pi}}=\sum_{i=1}^{4}\frac{\pm \rho_{ms}\cdot dh}{2\pi \mu_{0}r_{i}^{2}}\vec{r_{i}}$$

(3) $$\left\{ \begin{array}{l} dH_{P1}(z)=\frac{(-\rho_{ms})\cdot (z+h)dh}{2\pi \mu_{0}[{\left(x+a\right)}^{2}+{\left(y-b\right)}^{2}+{\left(z+h\right)}^{2}]}\\ dH_{P2}(z)=\frac{(+\rho_{ms})\cdot (z+h)dh}{2\pi \mu_{0}[{\left(x+a\right)}^{2}+{\left(y+b\right)}^{2}+{\left(z+h\right)}^{2}]}\\ dH_{P3}(z)=\frac{(-\rho_{ms})\cdot (z+h)dh}{2\pi \mu_{0}[{\left(x-a\right)}^{2}+{\left(y-b\right)}^{2}+{\left(z+h\right)}^{2}]}\\ dH_{P4}(z)=\frac{(+\rho_{ms})\cdot (z+h)dh}{2\pi \mu_{0}[{\left(x-a\right)}^{2}+{\left(y+b\right)}^{2}+{\left(z+h\right)}^{2}]} \end{array}\right.$$

where *r_i_* is the space vector from the magnetic charge element to the space point *P*(*x*, *y*, *z*); *μ*_0_ = 1.0 is the vacuum magnetic permeability; *a* is half of the spacing of the two parallel steel bars, and *z* is the lift-off height (LFH). Then, the normal component *H_p_*(*z*), expressed in Equation (4), can be obtained by the integral of Equation (3). The theoretical calculation results of Equation (4) are shown in Figure 7.

It can be seen from Figure 7 that as the corrosion extent increases (*h* increases), the amplitude of *H_p_*(*z*) increases continuously. While the *H_p_*(*z*) amplitude decreases with increasing z when the corrosion amount *Δm* is constant. The *H_p_*(*z*) curves has a zero-crossing intersection at the midpoint of the corroded region, and the peak-valley spacing equals the corrosion width 2*b*.

(4) $$\begin{array}{ll} H_{P}(z) & =\sum_{i=1}^{4}\int_{-h}^{0}dH_{Pi}(z)\\ & =\frac{\rho_{ms}}{4\pi \mu_{0}}(\ln\frac{{\left(x+a\right)}^{2}+{\left(y-b\right)}^{2}+{\left(z-h\right)}^{2}}{{\left(x+a\right)}^{2}+{\left(y-b\right)}^{2}+z^{2}}+\ln\frac{{\left(x+a\right)}^{2}+{\left(y+b\right)}^{2}+z^{2}}{{\left(x+a\right)}^{2}+{\left(y+b\right)}^{2}+{\left(z-h\right)}^{2}}\\ & +\ln\frac{{\left(x-a\right)}^{2}+{\left(y-b\right)}^{2}+{\left(z-h\right)}^{2}}{{\left(x-a\right)}^{2}+{\left(y-b\right)}^{2}+z^{2}}+\ln\frac{{\left(x-a\right)}^{2}+{\left(y+b\right)}^{2}+z^{2}}{{\left(x-a\right)}^{2}+{\left(y+b\right)}^{2}+{\left(z-h\right)}^{2}})\\ & =A\frac{\rho_{ms}}{4\pi \mu_{0}} \end{array}$$

## 3. Results and Discussion

### 3.1. Experimental Measurement Results of SMFL Signal

**Figure 8:**
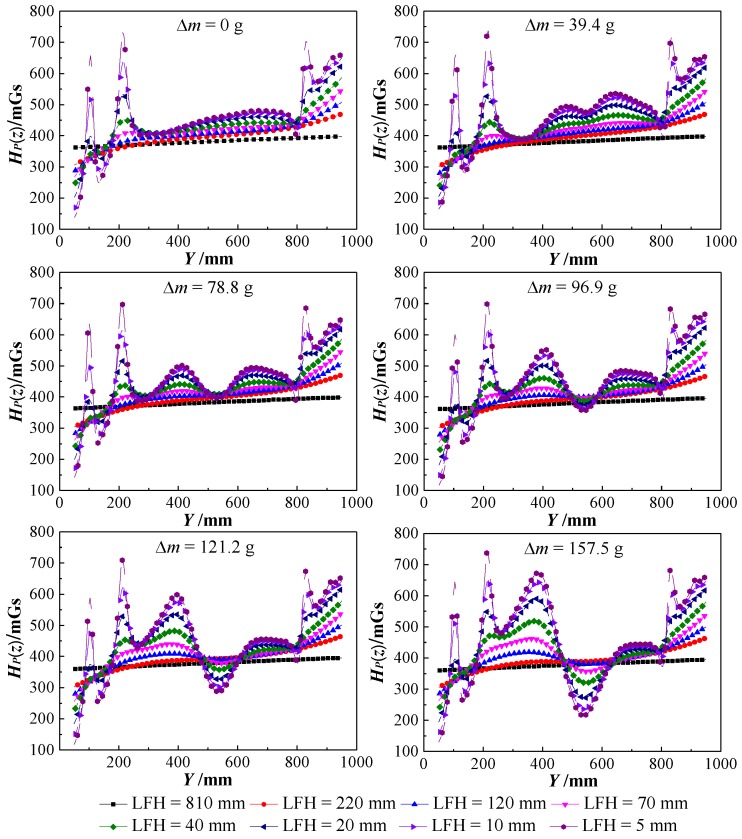
*H_P_*(*z*) curves of the nine tested beams with different *Δm*.

Figure 8 shows the normal component *H_P_*(*z*) curves of the nine tested beam SMFL signals with different corrosion amounts *Δm*, where *Y* = 400~550 mm corresponds to the corrosion region. The big spikes of amplitude outside of the corrosion region in Figure 8 are due to the effect of stirrups. As the corrosion amount *Δm* increased, the amplitude of the *H_P_*(*z*) curves in the corroded region increased, and then developed obvious peak-to-valley values and intersection; the smaller the LFH is, the larger the amplitude of *H_P_*(*z*) in the corroded region of the same *Δm*. The location and extent of the steel bars corrosion can be qualitatively determined by *H_P_*(*z*), apparently. All the experimental phenomena are consistent with the results of theoretical analysis, apparently.

In fact, the *H_P_*(*z*) shown in Figure 8 is a superposition of *H_B_*, *H_S_*, and *H_E_*. *H_B_* is the demagnetizing field of the steel bars, *H_S_* is the SMFL field originated from the corrosion region, and *H_E_* is the environmental magnetic field. *H_B_* and *H_E_* can be considered constant because the steel bars’ magnetization condition and the environment had not changed during the whole experimental process, which can be confirmed by the fact the *H_P_*(*z*) curves shown in Figure 8 did not significantly change within the uncorroded region. Therefore, the changes of *H_P_*(*z*) within the corroded region are mainly caused by the normal component *H_S_*(*z*) of the SMFL field that originated from the corrosion.

**Figure 9:**
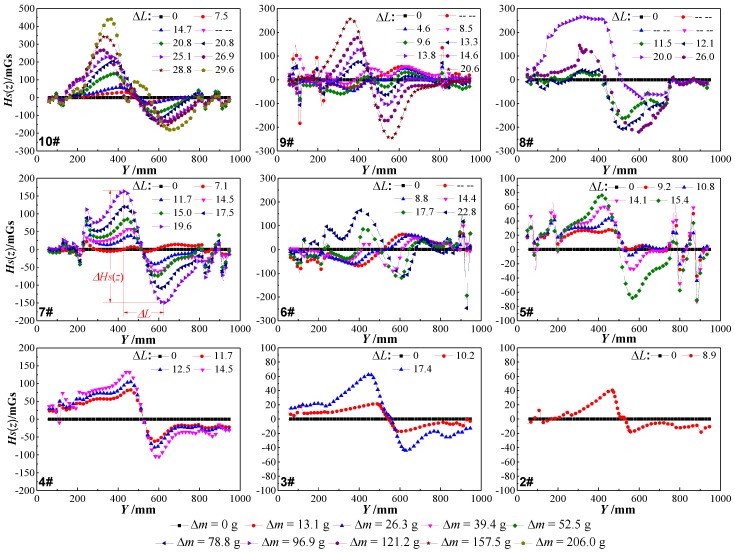
*H_S_*(*z*) curves of 2–10 tested beam with LFH = 5 mm.

For further analysis, *H_P_*(*z*) curves of each tested beam with the smallest LFH = 5 mm but different *Δm* were extracted from original *H_P_*(*z*) curves shown in Figure 8. The *H_P_*(*z*) curve with *Δm* = 0 g is defined as the background magnetic field (*H_B_* + *H_E_*), which is subtracted from each of the *H_P_*(*z*) curves to obtain the *H_S_*(*z*) curves originated from the corrosion region. Figure 9 shows the *H_S_*(*z*) curves of 2–10 tested beams (a small number of meaningless curves that do not affect the analysis results were deleted) and the Δ*L* (peak-valley spacing, unit: cm) corresponding to each *H_S_*(*z*) curve. As can be seen from Figure 9, the *H_S_*(*z*) curves are consistent with the theoretical analysis results, especially tested beam number four.

### 3.2. Analysis of SMFL Signal

In order to analyze the relationship between bending strength and SMFL signals of the corroded tested beams, the gradient *K* is defined. *K* is a direct and effective criterion for further description of the variation of SMFL signals, which is given by Equation (5):

(5) $$K=\left|\frac{\Delta H_{S}(z)}{\Delta L}\right|$$

where *ΔH_S_*(*z*) is the difference in the values of peak and valley *H_S_*(*z*) of the corroded area and *ΔL* is the peak-valley spacing of *H_S_*(*z*) curves, which are shown in Figure 9.

Table 3 shows the calculation parameters of gradient *K* of all tested beams. *ΔH_S_*(*z*)_Ea_ refers to the average value of the experimental *ΔH_S_*(*z*) with the same corrosion amount *Δm*; *ΔL_Ea_* is that of the experimental *ΔL* with the same corrosion amount *Δm*. *R* is the calculation cross-section remaining percentage of the corroded steel bars based on the uniform corrosion assumption, and the corrosion width 2*b* is assumed to be 15 cm when *R* is calculated. The conversion model corrosion depth *h*, integral value A, and magnetic charge density *ρ*_ms_ are the theoretical calculation parameters of Equation (4). Depth *h* is calculated according to R, and the calculation parameters of A are *x* = 0 cm, *y* = 7.5 cm, *z* = 3 cm, *a* = 2.5 cm, and *b* = 7.5 cm. Each *ρ*_ms_ is calculated according to its corresponding experimental *ΔH_S_*(*z*)_Ea_. *ΔH_S_*(*z*)*_T_* is the theoretical value of *ΔH_S_*(*z*). *K*_1_ and *K*_2_ refer to the theoretical gradient and the experimental gradient, respectively. The calculation formulas for all parameters are summarized in Equation (6):

(6) $$\left\{ \begin{array}{l} R=100\frac{15\times \pi \times 0.7^{2}\times 7.9\times 2-\Delta m}{15\times \pi \times 0.7^{2}\times 7.9\times 2}=100-\frac{\Delta m}{3.648}\\ h=1.4(1-0.01R)\\ \rho_{ms}=\frac{1}{2}\Delta H_{S}{\left(z\right)}_{Ea}\frac{4\pi \mu_{0}}{A}=\frac{2\pi \mu_{0}\Delta H_{S}{\left(z\right)}_{Ea}}{A}\\ \Delta H_{S}{\left(z\right)}_{T}=2\frac{\bar{\rho_{ms}}\cdot A}{4\pi \mu_{0}}=\frac{\bar{\rho_{ms}}\cdot A}{2\pi \mu_{0}}\\ K_{1}=\frac{\Delta H_{S}{\left(z\right)}_{T}}{2b}=\frac{\bar{\rho_{ms}}\cdot A}{0.3\pi \mu_{0}}\\ K_{2}=\frac{\Delta H_{S}{\left(z\right)}_{Ea}}{\Delta L_{Ea}} \end{array}\right.$$

**Figure 10:**
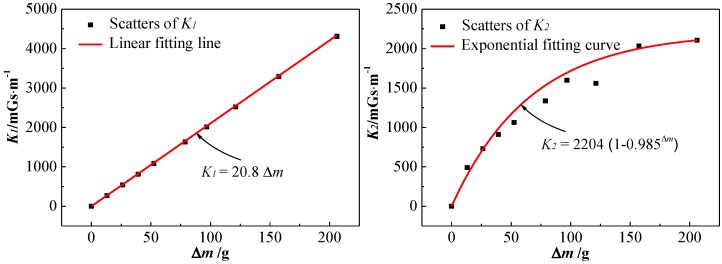
Diagram of relationship between *K* and *Δm*.

Figure 10 shows the relationship diagram between *K* and *Δm* according to Table 3. *K*_1_ is linearly related to *Δm*, the linear fitting equation is *K*_1_ = 20.8*Δm* with an R-squared value of 0.999. *K*_2_ is exponentially related to *Δm*, the exponential fitting equation is *K*_2_ = 2204 (1 − 0.985^*Δm*^) with an R-squared value of 0.946. The difference between *K*_1_ and *K*_2_ is mainly caused by the fact that the calculation of *K*_1_ is based on the constant assumed corrosion width, but the calculation of *K*_2_ is based on the continuous increasing actual corrosion width measured in the experiment.

### 3.3. Analysis of Bending Strength

Table 4 shows the bending strength *M* and its corresponding loss rate *S* of all tested beams, where the theoretical bending strength *M*_1_ and the experimental bending strength *M*_2_ are calculated according to the reinforced concrete structure design principle expressed in Equation (7):

(7) $$\left\{ \begin{array}{l} \alpha_{1}f_{c}bx=f_{y}A_{s}\\ M_{1}=\alpha_{1}f_{c}bx(h_{0}-\frac{x}{2})\\ A_{s}=0.01AR\\ M_{2}=\frac{1}{2}Fd \end{array}\right.$$

where *α*_1_ = 1.0 is the simplified calculation factor, *f_c_* = 14.3 MPa is the axial compressive design strength of the concrete, *b* = 100 mm is the section width of tested beams, *x* is the height of the concrete pressured zone, *f_y_* = 300 MPa is the tensile strength of the steel bar, for structure design, *A_s_* is the total cross-sectional area of corroded steel bars, *h*_0_ = 175 mm is the effective section height, *A* = 308 mm^2^ is the total cross-sectional area of uncorroded steel bars, *F* is the loading force measured in the experiment, and *d* = 0.62 m is the arm length of the force couple.

**Figure 11:**
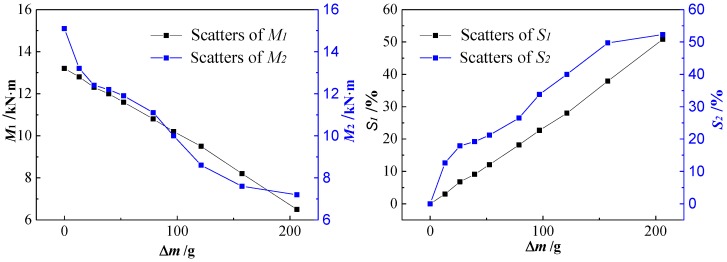
Diagram of the relationship between *M* and *Δm*.

Figure 11 shows the relationship diagram between *M* and *Δm* according to Table 4. It can be seen that both *M*_1_ and *M*_2_ decrease approximately linearly with the increase of *Δm*, especially *M*_1_. The values of *M*_2_ are not much different from that of *M*_1_, which indicates that the results of the experiment are consistent with that of the theoretical calculation. As for the loss rate *S*, *S*_1_ is positive linearly related to *Δm*, but *S*_2_ is more inclined to increase exponentially with the increase of *Δm*. The difference, mainly caused by the continuous increasing actual corrosion width in the experiment, leads to the cross-section of the steel bar being reduced more and more slowly with the same increment of *Δm*. Then, *M*_2_ decreased more and more slowly and *S*_2_ increased more and more slowly with the increase of *Δm*, which led to *S*_2_ increasing more and more slowly.

### 3.4. Analysis of Relationship Between SMFL Signal and Bending Strength

**Figure 12:**
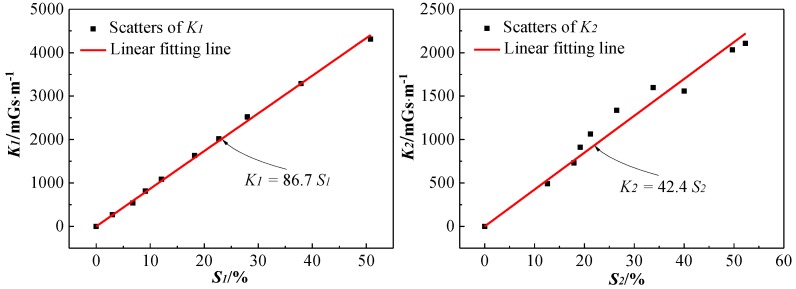
Scatters and linear fitting line of *S-K*.

According to the above analysis, both *K* and *S* increase monotonically with the increase of *Δm*. For evaluating the relationship between SMFL signals and the bending strength of the corroded reinforced concrete beams, scatters and the linear fitting line of *S-K* are shown in Figure 12. It can be seen that with the increase of *S*_1_ and *S*_2_, both *K*_1_ and *K*_2_ increase approximately linearly. The fitting function of the theoretical data is *K*_1_ = 86.7*S*_1_, with an R-squared value of 0.998, and that of the experimental data is *K*_2_ = 42.4*S*_2_, with an R-square value of 0.964. 

Compared with the theoretical data, the experimental data is more discrete and its gradient grows more slowly. In addition to being affected by the corrosion width, this difference may originate from the fact that some factors are ignored in the idealized theoretical analysis. Such as the magnitude and the distribution of magnetic charge, meaning that the shape of the corrosion notch will be affected by nonuniform corrosion or corrosion expansion stress.

However, in general, the variation and distribution laws of *S*-*K* obtained from the experiment are consistent with that of the theoretical analysis, which shows that the gradient *K* is a reliable and effective indicator for evaluating the residual bending strength of corroded reinforced concrete beams.

## 4. Conclusions

In this work, the experiment details of 10 corroded reinforced concrete tested beams based on micromagnetic sensors are introduced, followed by the derivation of the formulas of *H_P_*(*z*) (*H_S_*(*z*)) based on the magnetic dipole model. Finally, both the experimental data and theoretical data were analyzed. The following conclusions can be drawn:

(1) The experimental *H_S_*(*z*) curves of all tested beams are consistent with the theoretical calculation results of the magnetic dipole model, both the location and extent of the steel bars’ corrosion can be qualitatively determined using *H_S_*(*z*);

(2) The bending strength loss rate *S* of all the tested beams are approximately linearly related to the gradient *K* of *H_S_*(*z*), thus the residual bending strength of the corroded reinforced concrete beams can be evaluated based on the gradient *K* of *H_S_*(*z*).

This paper lays the foundation for the experimental study of the relationship between the bending strength and the SMFL signals of corroded reinforced concrete beams. A simpler, low-cost, and more efficient new method for evaluating the residual bending strength of corroded reinforced concrete beams based on the micromagnetic sensor is also proposed.

## Figures and Tables

**Table 1:** Material parameters of tested beam.

**Item**	**Label**	**Material**	**Cement**	**Water**	**Sand**	**Stone**	**Standard Strength (Mpa)**	**Design Strength (Mpa)**
Concrete	C30	Unit dosage (kg/m^3^)	461	175	512	1252	f_ck_ = 20.1	f_cd_ = 14.3
		weight ratio	1	0.38	1.11	2.72	f_tk_ = 2.01	f_td_ = 1.43
**Item**		**Reinforcement**					**Yield Strength (Mpa)**	**Design Strength (Mpa)**
Steel bars		Tensile bars: 2Φ14; Stirrup: Φ8@100					f_yk_ = 335	f_yd_ = 300

**Table 2:** Corrosion parameters of all tested beams.

Parameter	No.									
	1#	2#	3#	4#	5#	6#	7#	8#	9#	10#
Corrosion current/A	1.05	1.05	1.05	1.05	1.05	1.05	1.05	1.05	1.05	1.05
Corrosion time/h	0	12	24	36	48	72	96	120	156	504
*Δm*/g	0	13.1	26.3	39.4	52.5	78.8	96.9	121.2	157.5	206.0

**Table 3:** The calculation parameters of gradient *K*.

Parameter	*Δm*/g									
	0	13.1	26.3	39.4	52.5	78.8	96.9	121.2	157.5	206.2
*ΔH_S_*(*z*)_Ea_/mGs *ΔL*_Ea_/cm *R*/% h/cm A *ρ*_ms_/mGs *ΔH_S_*(*z*)*_T_*/mGs *K*_1_/mGs·m^−1^ *K*_2_/mGs·m^−1^	0 - 100 0 0 - 0 0 0	44.6 9.1 96.4 0.050 0.037 7574 40.4 269.5 490.1	84.1 11.5 92.80 0.101 0.075 7046 81.0 540.0 731.3	120.4 13.2 89.2 0.151 0.112 6754 121.7 811.6 912.1	159.5 15.0 85.6 0.202 0.150 6681 162.6 1084 1063	230.0 17.2 78.4 0.302 0.225 6423 244.8 1632 1337	313.0 19.6 73.4 0.372 0.278 7074 302.1 2014 1597	350.6 22.5 66.8 0.465 0.348 6330 377.9 2520 1558	501.8 24.7 56.8 0.605 0.454 6945 493.2 3288 2032	623.4 29.6 43.5 0.791 0.595 6583 646.5 4310 2106

**Table 4:** The bending strength *M* of all tested beams.

Parameter	No.									
	1#	2#	3#	4#	5#	6#	7#	8#	9#	10#
*Δm*/g *M*_1_/(KN·m) *S*_1_/% *F*/kN *M*_2_/(KN·m) *S*_2_/%	0 13.2 0 48.7 15.1 0	13.1 12.8 3.0 42.6 13.2 12.6	26.3 12.3 6.8 40.0 12.4 17.9	39.4 12.0 9.1 39.4 12.2 19.2	52.5 11.6 12.1 38.4 11.9 21.2	78.8 10.8 18.2 35.8 11.1 26.5	96.9 10.2 22.7 32.3 10.0 33.8	121.2 9.5 28.0 27.7 8.6 40.0	157.5 8.2 37.9 24.5 7.6 49.7	206.0 6.5 50.8 23.2 7.2 52.3
